# Identification and assessment of alleles in the promoter of the *Cyc‐B* gene that modulate levels of β‐carotene in ripe tomato fruit

**DOI:** 10.1002/tpg2.20085

**Published:** 2021-02-18

**Authors:** Caleb J. Orchard, Jessica L. Cooperstone, Elisabet Gas‐Pascual, Marcela C. Andrade, Gabriel Abud, Steven J. Schwartz, David M. Francis

**Affiliations:** ^1^ Department of Horticulture and Crop Science The Ohio State University OARDC, 1680 Madison Ave. Wooster OH 44691 USA; ^2^ Department of Food Science and Technology The Ohio State University 1739 N. High St. Columbus OH 43210 USA; ^3^ Present address: Department of Biochemistry and Molecular Biology University of Georgia 120 E. Green Street Athens GA 30602 USA; ^4^ Deparment of Biology Universidade Federal de Lavras Campus Universitário Lavras Minas Gerais 37200‐000 Brazil

## Abstract

Novel diversity may be mined from databases and de novo sequencing, but functional characterization remains a limiting step to identifying new alleles. Classical breeding approaches augmented by marker‐assisted selection offer a means to rapidly assess the function of new variation in coding or regulatory regions to modulate traits. We used the *Cyc‐B* gene (*B*) of tomato (*Solanum lycopersicum* L.) for a proof of concept because of its role in the production of β‐carotene, a provitamin A carotenoid with importance to human nutrition. We measured carotenoid content in vintage and contemporary varieties and the profiles had a range of β‐carotene from 0.2 to 4.06 mg 100 g^−1^ fresh weight. We characterized variation in *B* from 84 sequences recovered from public databases and from an additional 29 high β‐carotene tomato, *S. galapagense* S. C. Darwin & Peralta, and *S. cheesmaniae* (L. Riley) Fosberg accessions. Thirteen unique haplotypes across 1600 bp of sequence 5′ to the first ATG were identified with 11 occurring in high β‐carotene accessions we sequenced, and additional haplotypes were identified in public data. Phylogenetic analysis suggested that the alleles in high β‐carotene varieties were derived from wild species. Association analysis suggested two single nucleotide polymorphisms (SNPs) as the most likely causes of high β‐carotene, presumably through their influence on transcription of *B* that is elevated in ripening fruit. A marker‐assisted backcross breeding scheme leveraging SNPs for background genome selection was used to rapidly develop germplasm resources containing different alleles of *B* in a uniform genetic background. Evaluation demonstrated that distinct promoter haplotypes function as different alleles that can be used to modulate the levels of β‐carotene in tomato.

## Core Ideas

- The region 5′ to *Cyc‐B* is diverse with distinct haplotypes among high β‐carotene tomato.
- *Cyc‐B* haplotypes found in vintage and contemporary varieties are from wild species.
- SNPs shared between the green‐fruited clade and the Galápagos islands are likely functional.
- Marker‐assisted backcrossing was used to rapidly introgress natural variation for assessment.
- Fine‐tuning of β‐carotene content in tomato is possible using natural allelic variation.

**Abbreviations**
PCR: polymerase chain reaction
SNP: single nucleotide polymorphism
TGRC: Tomato Genetics Resource Center

## 1 INTRODUCTION

A challenge to crop improvement is the identification or development of new genetic variation. Traditional breeding approaches rely on identifying phenotypic variation, characterizing the underlying genetic variation, and incorporating new alleles into improvement programs. An alternative approach involves identifying natural variation at the sequence level and assessing whether this new variation imparts novel phenotypes. Finally, gene editing approaches create new variation in the hopes of modifying traits. The selection of novel alleles with superior phenotypes based on sequence comparison remains problematic without more complete information about the structural or regulatory consequences of variation combined with methods to rapidly assess the phenotypic consequences of new alleles.

Recent interest in selecting tomato (*Solanum lycopersicum* L.) varieties with diverse colors has been driven by the goals of market diversification and improved nutritional quality. Tomato fruit, with pigments ranging across the color spectrum, are routinely visible at markets and grocery stores. The predominant pigments in tomato fruit are carotenoids, which play an important role in the biology of plants and in the case of β‐carotene, provide an essential nutrient to animals that consume them. Carotenoids aid in light harvesting and photoprotection. They are also responsible for colorful pigmentation in flowers, which attracts pollinators, and in fruit, which entices animals that then disperse seed. Considerable documentation is available concerning the health benefits of carotenoids in the human diet (Johnson, 2002; Rao & Rao, 2007). The orange pigment β‐carotene is the most potent form of provitamin A and plays a role in cellular development and immune function (Grune et al., 2010). Other carotenoids, such as lutein, may protect against macular degeneration (Armoza et al., 2013). All‐*trans*‐lycopene is a red pigment in tomato whose consumption is associated with a reduced risk for prostate cancer, among other diseases (Er et al., 2014). Tetra‐*cis*‐lycopene is of interest because of its increased bioavailability compared with all‐*trans*‐lycopene (Cooperstone et al., 2015; Hadley et al., 2003; Unlu et al., 2007b; Unlu et al., 2007a)

Tomato provides a model for studying carotenoid biosynthesis because of changes in fruit color during ripening and an abundance of natural variation affecting color (Bramley, 2002; Hirschberg, 2001). High β‐carotene content in tomato is due to the *Beta* allele (*B*) of the *Cyc‐B* gene (Lincoln & Porter, 1950; Tomes et al., 1956; Tomes et al., 1954). Instead of primarily accumulating the red pigment *trans*‐lycopene, fruit from *Beta* plants accumulate β‐carotene and appear orange when ripe. The *Beta* allele *B* has been characterized using classical genetics and was described as a semidominant allele on chromosome 6 (Lincoln & Porter, 1950; Stommel & Haynes, 1994; Tomes et al., 1954). Reports describe *B* originating from the green‐fruited *S. habrochaites* S. Knapp & D.M Spooner (formerly *Lycopersicon hirsutum* Dunal) (Lincoln & Porter, 1950; Tomes et al., 1954). Other sources of the *B* allele trace to *S. galapagense* S. C. Darwin & Peralta [formerly *L. cheesmaniae* L. Riley var. minor (Hook. f.) D. M. Porter], and several of the other green‐fruited wild relatives (Chmielewski, 1965; Lesley, 1943; Lesley, 1947; Miller & Tanksley, 1990; Rick, 1956; Ronen et al., 2000; Soost, 1956; Stommel, 2001). A number of vintage cultivars also contain high β‐carotene, though the origin of this variation has not been described. Older tomato inbred varieties have been referred to as vintage to distinguish them from the more colloquial term heirloom, which has multiple meanings (Sim et al., 2012b; Williams and Clair, 1993). Vintage cultivars are sometimes also referred to as historic varieties.

**Core Ideas**

- The region 5′ to *Cyc‐B* is diverse with distinct haplotypes among high β‐carotene tomato.
- *Cyc‐B* haplotypes found in vintage and contemporary varieties are from wild species.
- SNPs shared between the green‐fruited clade and the Galápagos islands are likely functional.
- Marker‐assisted backcrossing was used to rapidly introgress natural variation for assessment.
- Fine‐tuning of β‐carotene content in tomato is possible using natural allelic variation.

Molecular characterization showed that the *Beta* locus (*B*) encodes a chromoplast‐specific lycopene‐β‐cyclase (*Cyc‐B*) with homology to neoxanthin synthase (Bouvier et al., 2000). The *Beta* locus (*B*) is one of four alleles previously described for the *Cyc‐B* gene; the other three being *old‐gold* (*og*), *old‐gold‐crimson* (*og^c^*), and the wild‐type *b* allele (Ronen et al., 2000). Both *og* and *og^c^* are loss of function, frame‐shift mutations in the structural gene (Ronen et al., 2000). The high β‐carotene content imparted by *B* appears to be related to variation in the promoter region (Ronen et al., 2000), and additional polymorphisms were described in a 908‐bp region upstream of *Cyc‐B* from *S. habrochaites* (Dalal et al., 2010; Mishra et al., 2002). The coding sequence is conserved between plants with *B* and the wild‐type allele *b*, but the region 5′ to the gene in both *S. pennellii* Correll and *S. habrochaites* is polymorphic and is correlated with an increase in transcript production in tomato fruit (Dalal et al., 2010; Ronen et al., 2000). Polymorphism in the promoter region also suggests that sequence variation may be an effective tool for tracing different sources of the *B* allele in cultivated tomato.

The specific functional changes leading to high β‐carotene tomato remain unknown. The objective of this study was to identify additional variation, and possibly functional polymorphisms, in the region 5′ to the *Cyc‐B* gene. The sequence for over 440 tomato genomes have become available (The 100 Tomato Genome Sequencing Consortium et al., 2014; Lin et al., 2014; The Tomato Genome Consortium, 2012), providing a resource to identify and examine allelic variation. With enormous quantities of sequence data now available for crop improvement, the question of how best to use existing allelic variation to enhance crops becomes relevant. The aim of this work was to provide a model to rapidly identify new genetic variation based on sequence, to introgress that variation into an elite background, and then assesses the effect of that variation. We hypothesized that the diverse sources of wild species used in breeding have contributed sequence variation to high β‐carotene cultivars and that novel sequence variation could be used to distinguish different sources of the *Cyc‐B* promoter. Here we describe carotenoid profiling of orange‐fruited tomato varieties, sequence diversity across 1,600‐bp 5′ to the *Cyc‐B* gene in high β‐carotene varieties and wild accessions, and use genetic analysis to identify polymorphisms associated with high β‐carotene content in tomato. We used backcross breeding with selection for diverse *B* haplotype sources and background genome selection in order to rapidly create near‐isogenic resources and assess the effect of sequence variation on carotenoid profiles. Background genome selection refers to the selection of the recurrent parent genome using molecular markers during backcross breeding (Servin & Hospital, 2002).

## 2 MATERIALS AND METHODS

### 2.1 Plant materials

Tomato seeds from academic and commercial sources were included in the experiments. Twenty of the 29 tomato varieties included in the carotenoid profiling were donated by the Tomato Genetics Resource Center (TGRC) in Davis, CA, or purchased from Tomato Grower's Supply Company in Ft. Myers, FL. The nine remaining varieties originated from both academic and commercial sources, including seeds that were kindly provided by Jay W. Scott and Sam Hutton of the Gulf Coast Research and Education Center at the University of Florida, Gainesville. In the sequencing collection of 27 accessions, we included tomato varieties exhibiting the *Beta* phenotype based on carotenoid profiles or containing the *B* allele from distinct genetic sources. Seeds for a Galápagos Islands tomato core collection consisting of six *S. cheesmaniae* (L. Riley) Fosberg and eight S. *galapagense* accessions were donated from the TGRC. The accessions of S. *cheesmaniae* (LA0531, LA1039, LA1406, LA1407, LA1409, and LA1412) and S. *galapagense* (LA0438, LA0483, LA0526, LA1044, LA1136, LA1141, LA1401, and LA1410) display the orange‐fruited *B* phenotype. LA0716 (*S. pennellii*) is a green‐fruited accession from the TGRC and was used to clone the *B* allele (Ronen et al., 2000). ‘Jaune Flamme’ tomato is an orange‐fruited variety from France with high β‐carotene content and frequently described as an heirloom (Kingsolver et al., 2008). 97L97 is an orange‐fruited processing tomato variety developed by introgressing the *B* allele from *S. galapagense* accession LA0317 into tomato cultivar Ohio 8245 (Stommel, 2001). Fla. 456 and Purdue 89‐28‐1 are orange‐fruited lines. Fla. 456 contains the *B* allele from *S. chilense* (Dunal) Reiche; Purdue 89‐28‐1 originated from Dr. Ed Tigchelaar's breeding program at Purdue University, with *B* derived from an unknown source.

We profiled 29 new accessions for carotenoids and combined these profiles with work from previous studies (De Nardo et al., 2009; Rubio‐Diaz et al., 2011; Rubio‐Diaz et al., 2010). Populations used for carotenoid profiling and Sanger sequencing were distinct, though some accessions overlapped between experiments. A total of 27 tomato accessions were sequenced by Sanger sequencing. Sequences from the hybrid ‘Sungold’ appeared to be heterozygous and therefore were not included in the alignments. The sequences for the remaining 26 accessions were compared with five publicly available sequences consisting of references ‘Heinz 1706’ (*S. lycopersicum*) and LA1589 (*S. pimpinellifolium* L.) as well as sequences from the three orange‐fruited *S. galapagense* accessions (LA0483, LA1044, LA1401) (The 100 Tomato Genome Sequencing Consortium et al., 2014). These 31 accessions were included in the sequencing alignments.

Two populations were used for association analysis. The first was a population of 84 individuals consisting of the 31 accessions included in the sequencing alignment as well as accessions from the SolCAP tomato collection (Sim et al., 2012a; 2012b) for which both data on lycopene and β‐carotene were available (De Nardo et al., 2009; Rubio‐Diaz et al., 2010, 2011) and *B* locus sequences could be inferred from pedigree. The second population consisted of the 31 accessions included in the Sanger sequencing alignments. This smaller population was a core set of the larger population of 84 in which *B* loci that were identical by descent based on pedigree were removed from the analysis in order to avoid over sampling the same mutations and confounding genetic replication with technical replication and thus inflating the degrees of freedom associated with error.

### 2.2 Carotenoid profiling

Two plants of each tomato line were grown in the greenhouse at temperatures between 21 and 30 °C. Ten mature fruit were harvested per line, five per plant, and pureed in a commercial blender (Waring Blender). Sample collection for field‐grown tomato fruit is described below. For each line or backcross sample, two 50‐ml tubes of fruit puree were stored at −80 °C for carotenoid extraction. Carotenoids were extracted from fruit puree samples as reported previously (Cooperstone et al., 2015; Kopec et al., 2014). Carotenoid concentrations are reported on a fresh‐weight basis. Varieties that contained high levels (>1 mg 100 g^−1^ fresh weight) of β‐carotene were used for DNA extraction and subsequent sequence analysis.

### 2.3 Sanger sequencing strategy

Leaf tissue was collected from seedlings for each line and stored at −80 °C for DNA extraction. A polymerase chain reaction (PCR) amplification strategy was used for sequencing. Genome sequences provided by the Tomato Genome Consortium for the red‐fruited tomato lines ‘Heinz 1706’ and LA1589 were searched using stand‐alone BLAST and ∼2,500 bp of sequence 5′ of the putative ATG start of translation for the chromoplast‐specific *Cyc‐B* gene on chromosome 6 was retrieved (Tao, 2010; The Tomato Genome Consortium, 2012). We developed three pairs of tiled primers, each amplifying ∼600 bp spanning 1,600 bp 5′ to the structural gene. Primer pairs (5′ to 3′) included (a) TGTTCCTAGGTTCGGTGAGC (F) and TAAGGTCTTCCGTGCCTGTT (R), (b) CAAAGCCTGTTGCCTTTCTC (F) and TAATTTTGCAGTTGGGCACA (R), and (c) AATATACCTGCCGTCCATGC (F) and TCTTCTCAAGCCTTTTCCATC (R).

Procedures for DNA extraction, PCR, and gel electrophoresis used in the analysis of these samples have been previously described (Robbins et al., 2009). Briefly, the PCR conditions were as follows: denaturation at 94 °C for 3.5 min, annealing at 56 °C for 30 s, and elongation at 72 °C for 45 s, repeated for 40 cycles. Amplified products were purified by precipitation using a 9:1 ethanol/sodium acetate mixture. Sequencing was performed at the Molecular and Cellular Imaging Center in Wooster, OH. Each amplicon was Sanger sequenced using the ABI Prism Sequencer (3100 × 1; Grand Island, NY), with sequence generated for each amplicon in both the forward and reverse directions.

### 2.4 Sequence alignments and marker development

Sequence data were quality checked and trimmed prior to alignment using the collection of programs in the STADEN software package (Bonfield et al., 1995). Contigs were assembled using PreGap4 and refined manually in Gap4. All new contigs generated in this study were submitted to the *GenBank* sequence database (see below).

Using the contigs generated in Gap4, sequences were aligned using MUSCLE (version 3.8) (Edgar, 2004). Using the alignment file from MUSCLE, a neighbor‐joining phylogenetic tree was created in MEGA 6.06 (Tamura et al., 2011) to visualize sequence divergence in the *Cyc‐B* promoter region. Briefly, the MUSCLE alignment file was loaded into MEGA 6.06 and the Tamura‐Nei model (Tamura & Nei, 1993), and 10,000 bootstrap replications were used in the phylogeny reconstruction.

We retrieved ∼2,000 bp of sequence 5′ of the putative start of the *Cyc‐B* gene from 84 tomato accessions published as part of The 100 Tomato Genome Sequencing Consortium (The 100 Tomato Genome Sequencing Consortium et al., 2014). Sequences were aligned in MUSCLE and the distance matrix was generated from the MUSCLE alignment file using the packages SeqinR and ape in R (Charif & Lobry, 2007; Edgar, 2004; Paradis & Schliep, 2019). Clustering was performed using the complete hierarchical clustering method with 1,000 bootstrap replications in the Pvclust package in R (R Core Team, 2013; Suzuki & Shimodaira, 2006).

### 2.5 Association analysis

Association analysis was conducted in R (R Core Team, 2013). Several approaches were used based on different assumptions about the independence of data, correction for population structure, and the distribution of trait data. Polymorphisms were scored relative to the initiation of transcription and insertion and deletion polymorphisms were treated as a single mutation.

Naïve association analysis was performed based on a simple linear model with no correction for population structure. For this analysis, levels of β‐carotene (mg g^−1^) were treated as a quantitative value and regressed on SNPs and insertion–deletions detected through sequencing. The statistical model was implemented as a naïve regressions using the lm function in R: **Y** *_n_* = **SNP** *_n_*_,_ *_k_* + Error*_n_*, where **Y** was the *n* × 1 vector of phenotypes with values averaged from high‐performance liquid chromatography data (see carotenoid profiling, above), and SNP was a series of presence or absence for *k* polymorphisms scored on *n* individuals included as a fixed effect. Analyses were also conducted using a nonparametric association model based on the Kruskal–Wallis test. For this analysis, β‐carotene levels were simply expressed as ‘high’ or ‘low’ because of the bimodal distribution of data.

Finally, an association analysis with a correction for population structure was also performed. For this analysis, we used the R package rrBLUP (Endelman, 2011). The model tested was implemented with the GWAS function in rrBLUP: **Y** *_n_* = **SNP** *_n_*_,_ *_k_* + **K** *_n_*_,_ *_n_* + Error*_n_*, where **Y** was as described above, **SNP** was a matrix of presence or absence for *k* polymorphisms scored on *n* individuals included as a fixed effect, and **K** was a matrix added as a random effect to correct for local structure in the *B* locus. The matrix **K** was derived from the MUSCLE alignment and represents a symmetrical matrix of pairwise DNA sequence identity along the *B* locus.

### 2.6 Backcross population development

Donor parents were LA3501, LA3502, and Jaune Flamme. The accessions LA3501, also known as IL 6‐2, and LA3502, also known as IL 6‐3, both contain the *B* allele from *S. pennellii* accession LA0716 introgressed into the M82 genetic background (Eshed & Zamir, 1995). The high β‐carotene variety 97L97, derives its *B* allele from *S*. *galapagense* (LA0317) and was developed by backcrossing to OH8245 (Stommel, 2001). The recurrent parent used for all alleles, OH8245, is a processing variety developed at the Ohio Agricultural Research and Development Center in Wooster, OH (Berry et al., 1991). Tomato varieties were grown in the greenhouse at temperatures between 21 and 30 °C. At each generation of recurrent backcrossing, progeny were crossed to OH8245.

### 2.7 Marker development

A molecular marker that distinguishes *S. pennellii*, Jaune Flamme, and red‐fruited *B* alleles was developed using sequence variation in a region 655 bp upstream from the initiation codon of the *B* gene. We designed a primer pair flanking a region with two unique insertion–deletion mutations. The primer pair (5′ to 3′) is as follows: CGTCTTAGGCTTGGGTTAGTTG (forward) and TGAGCTTCGCAACTTTCTCA (reverse). This PCR‐based marker was used to select for *B* alleles in both the heterozygous and homozygous states.

For the initial round of background selection, a custom panel of 96 SNP markers polymorphic between OH8245 and either the M82 genetic background or Jaune Flamme was chosen from the 7,720 SNP markers available on the SolCAP Infinium Array (Sim et al., 2012a; 2012b). Markers were selected on both arms of the 12 tomato chromosomes with a minimum distance of 5 cM between each SNP. The panel of 96 markers had 28 unique markers that were polymorphic between OH8245 and either recurrent parent background (Jaune Flamme or M82). Forty additional markers overlapped between all sources, totaling 68 polymorphic markers per source.

Using the same criteria as in the first marker design process, we developed a custom panel of 72 SNP markers for the second round of background selection. To further reduce the segments of donor parent genome, these markers were chosen in chromosomal regions that were not fixed after the first round of background selection. In this second panel, the M82 background had 18 unique polymorphic markers vs. OH8245, while the Jaune Flamme background had 29 unique markers. Twenty‐five additional markers were polymorphic between OH8245 and both backgrounds.

### 2.8 Marker‐assisted selection

Prior to genotyping, leaf tissue was collected from seedlings in the greenhouse and stored at −80 °C until DNA extraction. DNA extraction and PCR were performed according to procedures described in Robbins et al., 2009. Briefly, DNA extraction was performed in 96‐well cluster tubes (Corning, Inc.) using a CTAB procedure and a GenoGrinder (OPS Diagnostics). The PCR conditions included denaturation at 94 °C for 3.5 min, annealing at 56 °C for 30 s, and elongation at 72 °C for 60 s, repeated for 40 cycles. The PCR products were run on a 2% agarose gel at 180 V. We genotyped backcross one (BC_1_) progeny with the *B* PCR marker and selected individuals heterozygous for the *B* promoter allele. Heterozygous individuals and the parental controls were genotyped with a custom 96‐SNP marker array using a ligation–extension SNP assay as implemented on the Illumina BeadXpress platform (Illumina, Inc.). Following genotyping, data were quality checked for correct clustering using Genome Studio v2011.1 (Illumina, Inc.). Individuals containing the highest percentage recurrent parent genome were selected using the data.table v1.92 package in R for genotype calling and a script for allele frequency counting (Dowle and Srinivasan, 2017; R Core Team, 2013). Selected progeny were then subjected to a further round of backcrossing.

The BC_2_ progeny were again genotyped with the *B* PCR marker and individuals heterozygous for the B promoter were identified for a second round of background selection. These BC_2_ progeny and parental controls were genotyped with 72 SNP markers using Kompetitive Allele Specific PCR (http://www.lgcgenomics.com). Genotyping and quality analysis was performed by LGC Genomics in Beverly, MA. Selection was again performed using analysis in R to identify individuals with the highest proportion of OH8245 alleles.

### 2.9 Field trials

Following the first round of background selection, BC_1_ progeny with >83% recurrent parent markers and the *B* allele were self‐pollinated to produce BC_1_S_1_ plants. Multiple BC_1_S_1_ individuals were retained for the Jaune Flamme and LA0716 sources. These were maintained as separate families within each of the three donor populations. Within each population and family, BC_1_S_1_ plants were genotyped with the *B* PCR marker and separated into three classes relative to the state of the *B* locus: *BB*, *Bb*, and *bb*. Following genotyping, the BC_1_S_1_ plants were transplanted in the field. The BC_1_S_1_ plants were grown in the field in a randomized complete‐block design at two locations: Wooster, OH, and Fremont, OH. Each location contained two replications. Plants were grown in plots 3.05 m (10 feet) long, with 45.74‐cm (18 inches) spacing between plants. Most plots contained between eight and 10 plants. Replicated checks were included at each location. Check varieties included Jaune Flamme, M82, OH8245, Purdue 8928‐1, LA3501, LA3502, and 97L97. Ten ripe fruit were harvested from each plot. To avoid differences in ripeness, harvest was staged to collect fruit from the second cluster at full ripeness. Over a period of 4 wk, a total of five harvests at the Wooster location and seven at the Fremont location were performed to account for differences in plant maturity. Fruit from each plot were pureed in a Waring blender and stored in 50‐ml tubes at −20 °C until carotenoid extraction.

Following the second round of background selection, selected BC_2_ progeny were self‐pollinated and BC_2_S_1_ progeny were separated according to population and family, with population linking the selections back to the *B* source and family linking back to the BC_1_ selection. The BC_2_S_1_ plants were genotyped with the *B* PCR marker and further divided into *BB*, *Bb*, and *bb* allele classes. Plants were again grown in Wooster and Fremont, OH. The experimental design was the same as that used in the first year. Exceptions include dropping the LA3501 population as a result of extreme necrosis of plants observed in the first year of evaluation. Checks for the second year field trial included PS696 and the hybrid formed by crossing 97L97 and Purdue 8928‐1. Fruit sampling and carotenoid profiling were as described above.

### 2.10 Data analysis

An ANOVA was performed using a linear regression model to examine the effects of promoter source (population), BC_1_ selection (family), and *B* allele state (*BB*, *Bb, bb*) on β‐carotene content in tomato fruit. Analysis was performed using the lmer function of the lme4 package in the R statistical program (Bates, Mächler, Bolker, & Walker, 2015). The experimental model was as follows:

**y** *= u* + POP + FAM(POP) + Allele_B + Allele_B:POP + LOC + REP(LOC):YEAR + YEAR + Error

The variable **y** is a vector of phenotype values measured, specifically β‐carotene and lycopene content; *u* is the grand mean for the variable in question. All terms in the model were considered random. POP denotes population effect attributed to the source of the *B* allele (Jaune Flamme or LA0716). FAM(POP) is the effect attributed to family within population and is an indication of allele affects other than *B* that may be segregating. Allele_B is the effect resulting from having either the *B*, wild‐type *b*, or heterozygous allele state. The interaction Allele_B:POP is the effect of the allele in the population. LOC denotes the experimental location, while REP(LOC):YEAR is the term describing variation within a particular environment. YEAR denotes the year in which the experiment was conducted. Finally, Error is the residual variation unaccounted for in the experimental design. The proportion of variance explained was estimated from lme4 using the ‘Summary’ statement. Significance was determined for model effects by ANOVA using a fixed‐effects model containing the same variables as the random‐effects model.

### 2.11 RNA extraction and gene expression

Tomato fruits were harvested at four stages of ripening: mature green (MG), breaker (B), turning (B+3 d) and red‐ripe (B+7 d). Two fruits from each stage were taken, and slices from the bottom part of the pericarp were pooled together. Skin was removed and tissue was deep frozen in liquid N_2_. Frozen tissue was then ground to a fine powder with a mortar in liquid N_2_. Approximately 100 mg of ground tissue was used for RNA extraction with Trizol Reagent (Life Technologies). Frozen tissue was homogenized in 900 μl of Trizol and incubated 1 h at 4 °C. The aqueous phase (600 μL) containing the RNA was recovered after chloroform extraction, and RNA was precipitated by adding 0.5 vol of 0.8 M sodium citrate and 1.2 M sodium chloride and 0.5 vol isopropanol followed by incubation at 4 °C. After 15 min centrifugation at 12.000 *g* and 4 °C, pellets were washed twice with 75% EtOH and air‐dried. The RNA was suspended in 30–50 μl RNAse‐free water and stored at −80 °C. The RNA was quantified using a NanoDrop ND‐1000 spectrophotometer (NanoDrop Technologies) and quality was assessed by visualization after electrophoresis on agarose gels and EtBr staining. The RNA was then treated with DNAse I (DNA‐free kit, Life Technologies) and cDNA was synthesized from 1 μg DNAse‐treated RNA using RETROscript kit (Life Technologies). Gene expression was analyzed by real‐time PCR using the KAPA SYBR FAST qPCR Kit (KapaBiosystems) and quantified using an IQ5 cycler (Bio‐Rad Laboratories, Inc.). Expression was analyzed for *CYC‐B* (Solyc06g074240.3.1; using Forward 5′‐TCCTAAATACTGGCAAGGGT‐3′ and Reverse 5′‐TGTTTGAGCCATGTCCGAA‐3′). The expression control was *EF1* (Solyc06g005060.3.1; using Forward 5′‐ CCTCCGTCTTCCACTTCA‐3′ and Reverse 5′‐TCACACCAGTCTCAACGC‐3′).

## 3 RESULTS

We surveyed tomato fruit from a variety of seed sources based on descriptions of flesh color and carotenoid content in order to determine which tomato varieties contained β‐carotene as the predominant pigment. We quantified carotenoids for 29 varieties (Table 1). Nine varieties contained >1 mg fresh weight β‐carotene and were considered high β‐carotene accessions. The amount of β‐carotene within these nine varieties ranged from 1.60 to 4.06 mg 100 g^−1^ fresh wt.. Of the remaining samples, nine varieties were classified as primarily containing all‐*trans*‐lycopene. The cultivar Caro Red (LA2374) was unique in that it was classified as high β‐carotene (2.97 mg 100 g^−1^ fresh wt.) but also contained all‐*trans*‐lycopene (3.64 mg 100 g^−1^ fresh wt.) as the predominant carotenoid. Eleven varieties contained tetra‐*cis*‐lycopene as the predominant carotenoid, accounting for the orange color of these accessions. Among the high β‐carotene varieties, variation in levels suggested that either differences in genetic backgrounds or source of *B* might play a role in determining β‐carotene content.

**TABLE 1:** Carotenoid profiling by high‐performance liquid chromatography of 29 orange‐ and red‐fruited tomato accessions

Name	Accession code	Seed source^a^	Class	β‐carotene	All *trans*‐lycopene	tetra‐*cis*‐lycopene
				mg (100 g fresh weight)^−1^		
LA0316	LA0316	TGRC	High β	4.06	0.96	N/D^b^
862978	862978	Western Seed Americas Inc.		3.24	0.84	
‘Purdue 89‐28‐1’	Purdue 89‐28‐1	Harris Moran Seed Co.		3.19	0.94	
Fla. 456	Fla. 456	GCREC		3.06	1.62	
LA4380	LA4380	TGRC		3.00	2.63	
‘Caro Red’	LA2374	TGRC		2.97	3.64	
97L97	97L97	John Stommel, USDA/ARS		2.68	0.14	
‘Jaune Flamme’	SCT_0300	Tomato Grower's Supply		1.78	1.19	
‘Sungold’	N/A^c^	FedCo Seeds		1.60	1.08	
LA4421	LA4421	TGRC	Red	0.97	3.35	N/D
‘Purple Calabash’	LA2377	TGRC		0.79	8.04	
‘German Giant’	N/A	Tomato Grower's Supply		0.31	4.25	
‘Georgia Streak’	LA2969	TGRC		0.29	3.30	
OH8245	SCT_0116	OARDC		0.26	6.08	
M82	LA3475	OARDC		0.24	5.95	
‘Georgia Streak’	N/A	Tomato Grower's Supply		0.23	2.14	
OH7530	SCT_0021	OARDC		0.22	6.96	
BR03‐7264	BR03‐7264	OARDC		0.20	11.46	
‘Hawaiian Pineapple’	N/A	Tomato Grower's Supply	Tangerine	N/D	0.06	1.82
‘Kellogs Breakfast’	N/A	Tomato Grower's Supply			0.01	3.17
‘Kentucky Beefsteak’	N/A	Tomato Grower's Supply			0.08	3.37
‘Orange Strawberry’	SCT_0306	Tomato Grower's Supply			0.21	4.79
‘Persimmon’	N/A	Tomato Grower's Supply			0.06	3.15
‘Sunray F1’	N/A	Tomato Grower's Supply			0.04	7.90
‘Tangella’	N/A	Tomato Grower's Supply			0.14	3.37
‘Valencia’	N/A	Tomato Grower's Supply			0.03	3.19
‘Verna Orange’	N/A	Tomato Grower's Supply			0.04	4.40
‘Orange Minsk’	N/A	Tomato Grower's Supply			0.08	5.84
LA2971	LA2971	TGRC			0.12	2.69

^a^ TGRC, Tomato Genetics Resource Center, University of California, Davis; GCREC, Gulf Coast Research and Education Center at the University of Florida, Wimauma, FL; OARDC, Ohio Agricultural Research and Development Center, The Ohio State University, Wooster, OH; USDA–ARS, Genetic Improvement of Fruits and Vegetables Laboratory, Beltsville, MD. Commercial seed sources included Western Seed Americas, Plant City, FL; Harris Moran Seed Co. Modesto, CA; FedCo Seeds in Waterville, ME; and Tomato Grower's Supply in Fort Myers, FL.

^b^ N/D, not detected (below limit of detection).

^c^ N/A, not available;

**FIGURE 1:**
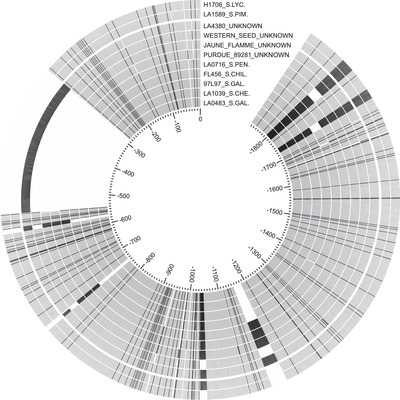
Variation in the DNA sequence directly 5′ to the *Cyc‐B* gene. Each ring represents a unique promoter haplotype indicated by a tomato variety or accession. Nucleotide differences, either insertion–deletion mutations or single nucleotide polymorphisms are shown in black. ‘0’ represents the start codon ATG at position 43,564,022 according to the Heinz 1706 reference genome SL4.0. Sequence alignment was created using MUSCLE version 3.8 (Edgar, 2004)

Sanger sequencing was performed for 27 tomato accessions comprised of nine high β‐carotene accessions, the green‐fruited *S. pennellii* LA0716 accession (source of high β‐carotene in LA3501 and LA3502), the tangerine cultivar Verna Orange, five red‐fruited accessions, as well as six *S. cheesmaniae* and five *S. galapagense* accessions with the *B* allele. These 27 sequences were compared with the references Heinz 1706 (*S. lycopersicum*) and LA1589 (*S. pimpinellifolium*) as well as recently published sequences from the three orange‐fruited *S. galapagense* accessions (LA0483, LA1044, LA1401) (The 100 Tomato Genome Sequencing Consortium et al., 2014). Contigs assembled from sequencing reads covered ∼1,600 bp 5′ from the first ATG in the chromoplast‐specific *Cyc‐B* gene. Sequence data showed that the promoter region of *Cyc‐B* was highly variable. Sequences from the hybrid Sungold appeared to be heterozygous and therefore were not included in the alignments. We observed 95 SNPs and 19 insertions–deletions relative to the Heinz1706 reference sequence (Figure 1). LA4380 contained a 258‐bp insertion, and we therefore sequenced over 1,850 bp for this accession.

**FIGURE 2:**
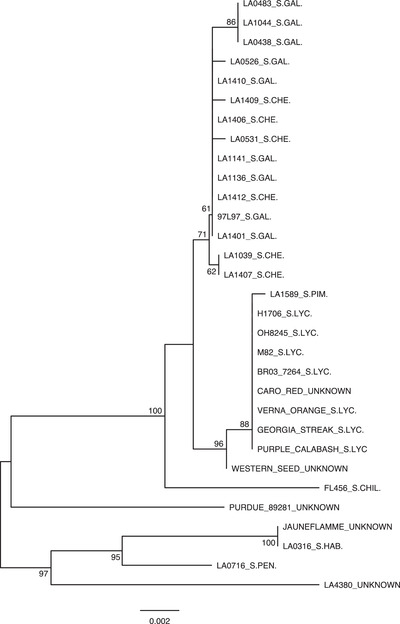
Phylogenetic tree of the *Cyc‐B* promoter region. Nodes indicate tomato varieties or accessions as well as the source of the *Cyc‐B* promoter. A neighbor‐joining tree was constructed using MEGA 6.06 (Tamura, Stecher, Peterson, Filipski, & Kumar, 2013). Ten thousand bootstrap replications were performed using the Tamura‐Nei model (Tamura & Nei, 1993) and bootstrap values are given for each branch

Alignments and phylogenetic trees developed from the sequence data were used to visualize the relationship between haplotypes. Thirteen distinct *B* promoter haplotypes were identified as a result of the clustering analysis (Figure 2). Eleven of these haplotypes occurred among accessions with high β‐carotene. Clustering also suggested clades separating the *B* promoter haplotypes along the classical subgenus *Eulycopersicon* and *Eriolycopersicon* split corresponding to red‐fruited and green‐fruited species. Accessions in which *B* promoter haplotypes originated from known green‐fruited species formed an outgroup basal to the red‐fruited sequences. The promoter sequence from Jaune Flamme clustered with promoter sequences derived from the green‐fruited wild species *S. habrochaites* and was identical to that of LA0316. The sequence for Purdue 89‐28‐1 also clustered with the green‐fruited clade. The Galápagos Islands tomato fruits formed an independent clade from both the red and green‐fruited species. The high β‐carotene variety 97L97, derives its *B* allele from *S. galapagense* (LA0317) (Stommel, 2001) and clustered in the Galápagos group. The variety Western Seed 862978 appeared to cluster more closely with the red‐fruited accessions, though independently of the larger group. The high β‐carotene cultivar Caro Red contained a *B* promoter allele that was identical to the majority of the red‐fruited accessions, suggesting an alternative mechanism for high β‐carotene in this variety. It appears that the majority of *B* alleles found in high β‐carotene varieties and accessions are derived from wild species.

**FIGURE 3:**
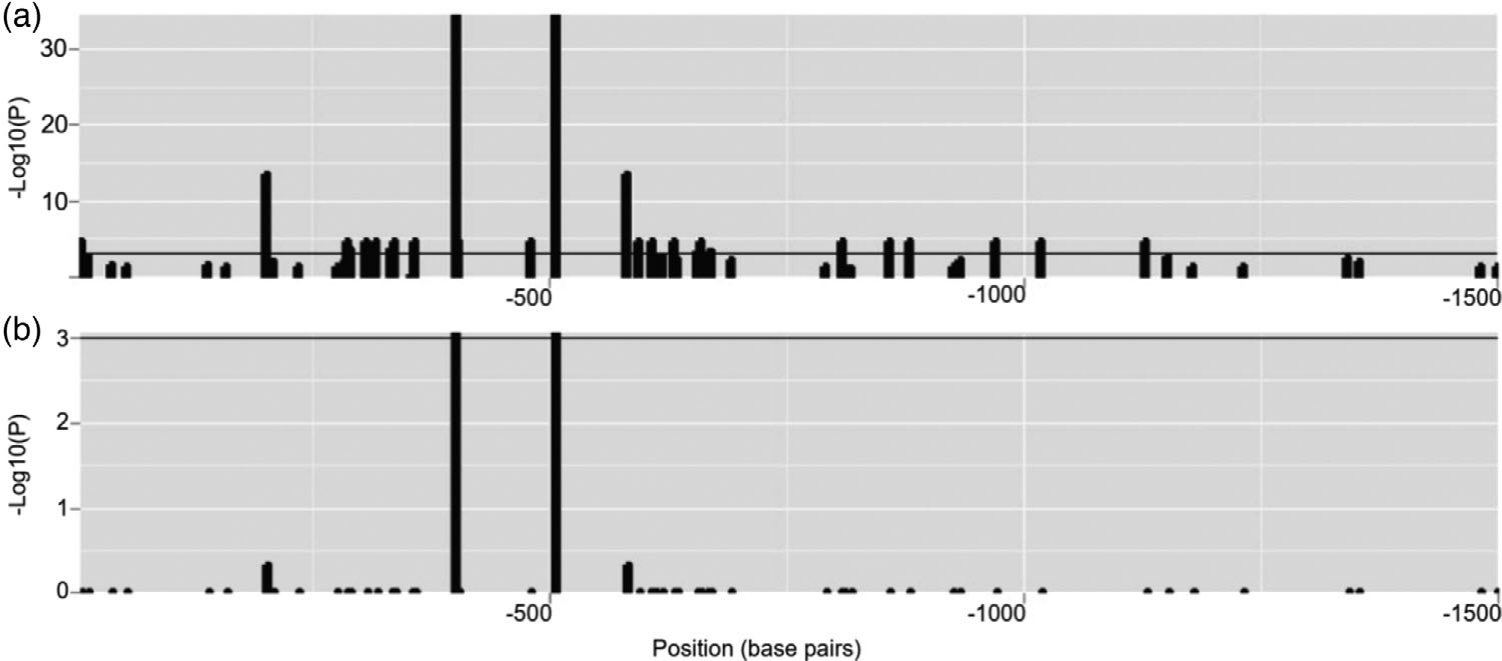
Putative functional single nucleotide polymorphisms (SNPs) within the *B* promoter identified by association analysis. Manhattan plot showing position of SNP or insertion–deletion variation relative to the *Cyc‐B* start codon (ATG = 0, position 43,564,022 according to the Heinz 1706 reference genome SL4.0ch06 vs. −Log10(P) based on association with β‐carotene levels in fruit. (a) Naïve regression with no correction for structure; (b) Association analysis using a correction for structure based on local sequence variation by converting the distance matrix from MUSCLE to a similarity matrix. Horizontal line represents *P* = .001. The two most significant SNPs are located at 401 bp, C (red) > T (high β‐carotene), and at 506 bp, G (red) > A (high β‐carotene) 5′ of the start codon. These positions are at 43,564,423 and 43,564,528 relative to the H1706 reference (SL4.0ch06)

Association analysis was conducted using 52 SNP or indel polymorphisms for which the minor allele frequency was greater than 0.05. Analysis based on the larger population (*n* = 84) and the smaller core set (*n* = 31) identified the same two SNPs as the most likely candidates for the functional mutations conferring high β‐carotene (Figure 3). The first SNP, C (red) > T (*Beta*) is located 401 bp upstream of the ATG (43564022 on the H1706 reference sequence (SL4.0ch06) at position 43,564,423. The second SNP, G (red) > A (*Beta*), occurs 506 bp 5′ to the putative *Cyc‐B* start codon, at position 43,564,528 on the H1706 reference sequence. These two SNPs occur in all the high β‐carotene varieties and accessions with the exception of Caro Red. The majority (36 of 52) of SNP or indel markers were significant at *P* < .01 using a naïve regression model on the full population for which both phenotypic values and sequence data were available. The SNPs at positions −401 and −506 bp were significant at *P* = 3.74 × 10^−35^. We explored more conservative analysis to adjust for both phenotypic distribution and substructure within the population. Using a Kruskal*–*Wallis test to account for a nonparametric distribution of phenotypic values, a high proportion of SNPs or indel markers were significantly associated with β‐carotene content, with 52 significant at *P* < .05 and 34 significant at *P* < .01. SNPs at position −401 and −506 were significant at *P* < 2.2 × 10^−16^. When the association model was corrected for local structure using the similarity matrix derived from sequence alignment, SNPs −401 and −506 bp were the only markers associated with β‐carotene content (*P* = 8.64 × 10^−4^). Because a large number of red‐fruited accessions derived the *B* locus by descent, we narrowed the population to a smaller core set in order to minimize the effect of treating identical alleles as separate genotypes in the analysis. Using the smaller core population, the SNPs at position −401 and −506 bp were significant at *P* = 4.32 × 10^−8^ with the Kruskal*–*Wallis model, *P* = 7.4 × 10^−10^ with the naïve regression and *P* = .001 in the *K*‐corrected model.

We used marker‐assisted backcrossing to accelerate the introgression of the *B* locus into a processing tomato background. We developed a PCR‐based molecular marker to distinguish alleles of the *B* promoter. The primer pair amplified a region 655 bp upstream of the *B* gene in which both *S. pennellii* and Jaune Flamme have different length deletions relative to the red cultivated varieties. The marker identified the deletion mutations CCTTGTTCGAGTTCCTTTATAAA in Jaune Flamme and GTCTAATACTCTAACTA in *S. pennellii* and allowed three alleles of *B* to be distinguished.

**FIGURE 4:**
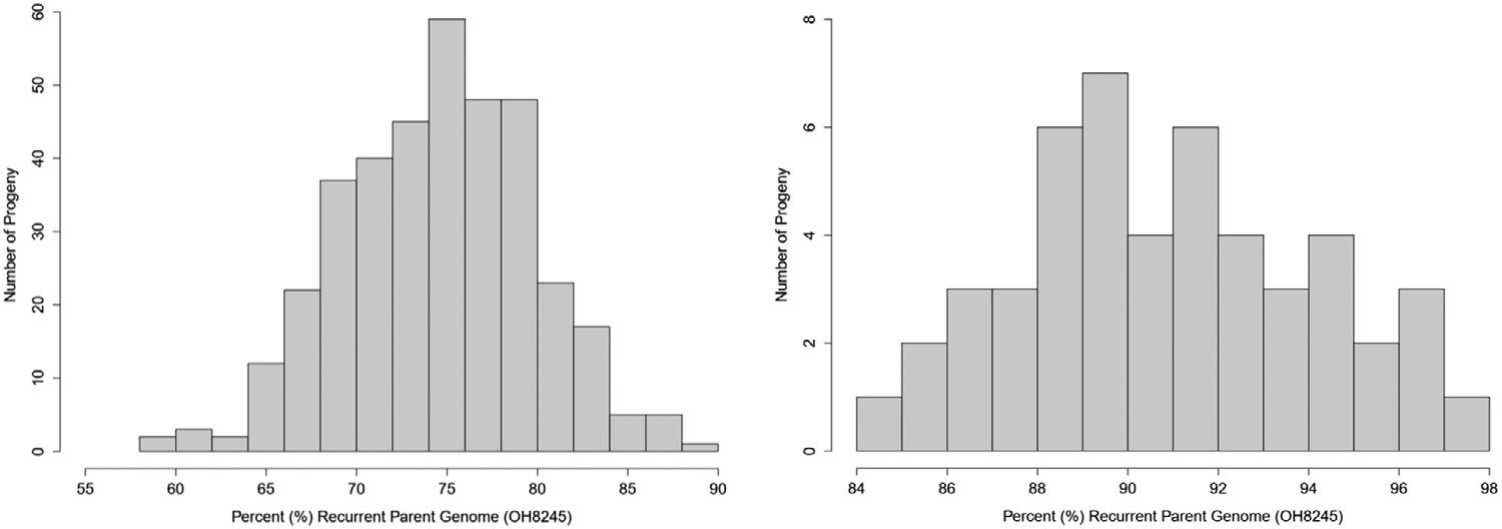
Distribution of progeny vs. percentage recurrent parent genome in the BC_1_ (left) and BC_2_ (right) generations. The *y* axis indicates the number of progeny. The *x* axis is the percentage of recurrent parent genome (OH8245). BC_1_ individuals containing between 83 and 88% recurrent parent genome were selected for further backcrossing and evaluation (BC_1_S_1_). BC_2_ individuals with at least 93% recurrent parent genome were selected for evaluation (BC_2_S_2_)

We genotyped 1,444 BC_1_ progeny from three populations with the *B* PCR marker and selected 384 individuals heterozygous for the *B* promoter allele. The three populations were derived from donor parents Jaune Flamme, LA3502, and LA3501 backcrossed to recurrent parent OH8245. Each population contained 137, 140, and 94 individuals heterozygous for *B*, respectively. These 384 heterozygous plants were genotyped with a custom set of 96 SNP markers to select those individuals containing the highest percentage of recurrent parent genome. The 96 SNPs were distributed across the genome with an average of eight markers per chromosome, and minimum–maximum range of six to 10 markers per chromosome. All three populations averaged ∼75% recurrent parent genome based on the markers included in the assay (Figure 4). The Jaune Flamme population averaged 75.1%, the LA3501 population averaged 74.9%, and the LA3502 population averaged 73.7% recurrent parent genome. Fourteen individuals, four to five from each donor, with between 83–88% recurrent parent alleles, were selected for further backcrossing. These individuals represent plants that contain an approximate proportion of recurrent parent alleles expected at the BC_2_ stage.

The 14 selected BC_1_ plants were backcrossed to OH8245 to produce BC_2_ progeny. Forty‐nine individuals heterozygous for the *B* promoter were identified following PCR screening of 95 BC_2_ plants with the *B* PCR marker. For the second round of background selection, we chose 72 SNP markers located in areas of the genome that were not yet fixed after the first round of background selection. Marker number averaged six SNPs per chromosome, but additional SNPs were selected on chromosomes with multiple segments that were not fixed according to the BC_1_ stage marker data. Nineteen markers were monomorphic in the BC_2_ progeny and were removed from the dataset. The remaining 51 markers were used for background genome selection. One individual was discarded because of missing data, reducing the dataset to 48 individuals. The total population of 48 individuals averaged 90.9% recurrent parent genome (Figure 4). The average proportion of recurrent parent genome for the individual populations was 89.3, 92, and 92.5% for Jaune Flamme, LA3501, and LA3502, respectively. These averages are near the expected range of 91.5–94% recurrent parent genome based on the BC_1_ selections. Background genotyping identified six BC_2_ plants, two for each population (Jaune Flamme, LA3501, and LA3502) containing between 93 and 98% OH8245 genetics.

Field trials consisting of segregating populations for the *B* promoter were conducted to determine if the source of the *B* promoter and the state of the *B* allele affected β‐carotene production in the fruit. Selected BC_1_S_1_ families were planted in two locations in the first year and BC_2_S_1_ families were planted in the second year. The relationship between BC_1_S_1_ families and BC_2_S_1_ families was maintained as part of the experimental design as family within promoter. Severe necrosis was observed in plants that were homozygous for the introgression from LA3501 in BC_1_S_1_ trials, and all plots from the LA3501 population were excluded from analyses and selections were dropped prior to second year field trials.

Variance partitioning of β‐carotene levels in ripe fruit samples, quantified using high‐performance liquid chromatography, revealed that allele state explained the largest proportion of total variation in β‐carotene content (Table 2). For field data combining both trials, the allele state was responsible for 52.1% (*P* < .001) of the total variation for β‐carotene content. The source of the *B* promoter explained 7.7% of the total variation for β‐carotene content (*P* < .001). The effect of the allele within each promoter source explained 16.44% (*P* < .001) of the total variation for β‐carotene content. Family within promoter source explained <1% of the total variation for β‐carotene content, but was significant (*P* = .002). Year was also significant (*P* = .01), but explained <1% of the total variation for β‐carotene. Location, replication within location × year and the interaction terms location × year and population × year did not explain a significant portion (*P* > .05) of the total variation for β‐carotene. Residual error explained 11.1% of the variation for β‐carotene.

**TABLE 2:** Sources of variation for β‐carotene content in ripe tomato fruit over 2 yr

Sources of variation^a^	Variance	SD	Total variance
			%
Allele	1.09	1.05	52.13**
POP	0.16	0.40	7.70**
LOC	0.00	0.00	0.00
YEAR	0.02	0.12	0.70*
FAM(POP)	0.01	0.12	0.65**
Allele_B:POP	0.35	0.59	16.44**
POP:YEAR	0.01	0.07	0.22
LOC:YEAR	0.00	0.00	0.00
REP(LOC):YEAR	0.23	0.48	11.07
Residual	0.23	0.48	11.09

^a^ Allele is the state of *B* (*BB*, *Bb*, *bb*); POP denotes the source of the *B* promoter (Jaune Flamme, LA3502); LOC denotes location (Fremont or Wooster); YEAR denotes the year of field trials; FAM(POP) denotes a specific cross within each *B* source population; Allele_B:POP is the interaction between the state of the *B* allele and the source of the *B* allele; POP:YEAR is the interaction between the source of the *B* allele and the year of field trials; LOC:YEAR denotes the interaction between location and the year of field trials; REP(LOC):YEAR is the effect of replication within each location, across each year of field trials.

* Significant at *p* = .01.

** Significant at *p* = .001.

**FIGURE 5:**
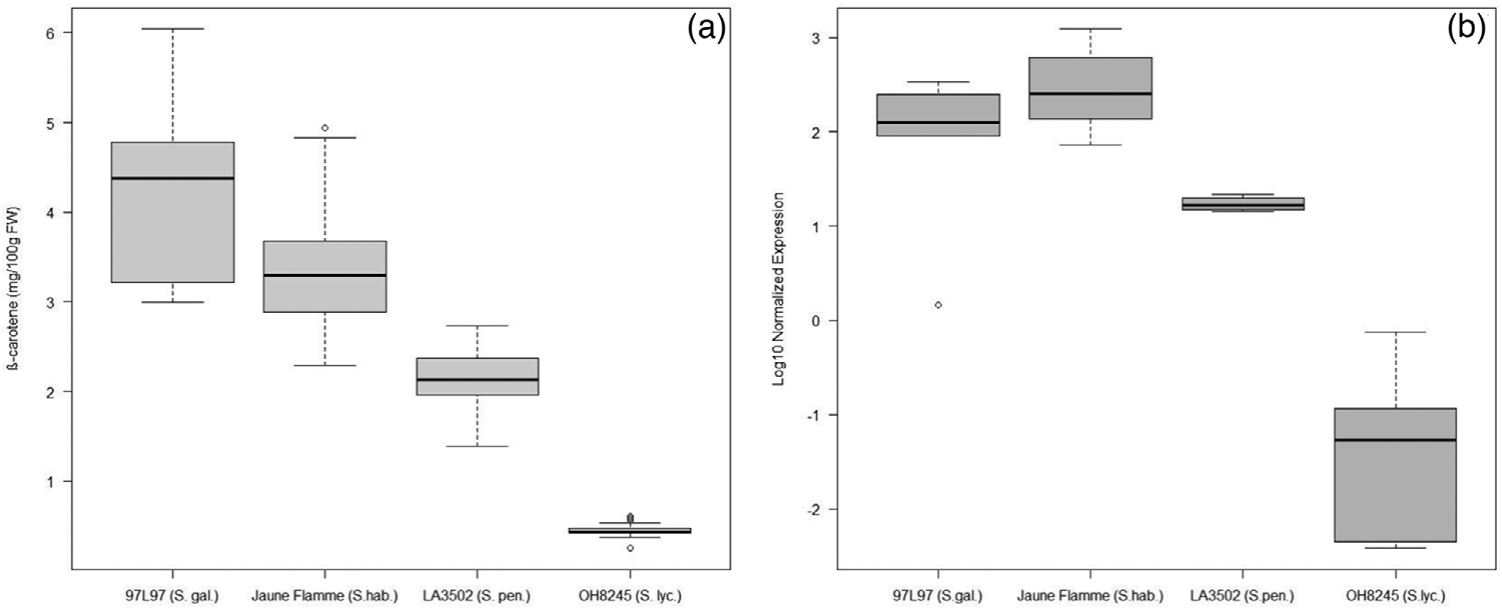
β‐carotene content and *CYC‐B* mRNA expression in tomato fruit according to the source of the *B* allele. (a) *y* axis indicates the level of β‐carotene in milligrams per 100 g fresh weight. The *x* axis is the donor source of the *B* allele. Boxes indicate levels of β‐carotene in ripe tomato fruit from BC_1_S_1_ and BC_2_S_2_ families in the OH8245 genetic background and homozygous for the *B* allele. (b) *y* axis indicates the level Log_10_–transformed values of *CYC‐B* mRNA normalized relative to *Elongation Factor 1* (*EF1*) for fruit that were at the breaker + 3‐d maturation stage. The *x* axis is the donor source of the *B* allele. Boxes indicate *CYC‐B* mRNA expression from BC_2_S_2_ selections in the OH8245 genetic background and homozygous for the *B* allele. The source of the *B* allele for each is *S. galapagense* (S. gal.), *S. habrochaites* (S. hab.), and *S. pennellii* (S. pen.). OH8245 contains the common red‐fruited *b* allele from *S. lycopersicum* (S. lyc.)

Plants with wild‐type (*bb*) allele averaged 0.54 mg 100 g^−1^ β‐carotene, while plants that were homozygous for *B* had on average 2.79 mg 100 g^−1^ β‐carotene. Overall, when the *B* allele was in the heterozygous state, average β‐carotene levels (2.16 mg 100 g^−1^) nearly reached those of the homozygous *B* plants. We compared plants with the *B* allele introgressed into OH8245 from three donor sources (*S. pennellii*, Jaune Flamme, and *S. galapagense*). Backcross families with the *B* allele from LA3502 (LA0716) averaged 2.12 mg 100 g^−1^ β‐carotene, while families with the Jaune Flamme allele of B averaged 3.36 mg 100 g^−1^ β‐carotene, and those with the 97L97 allele (LA0317) averaged the 4.15 mg 100 g^−1^ β‐carotene (Figure 5a). These results demonstrate that the alleles of *B* inherited from *S. pennellii*, Jaune Flamme, and *S. galapagense* are able to modulate the level of β‐carotene in fruit.

Differences in *CYC‐B* mRNA expression were detected among lines containing different *B* alleles. The distribution of expression data suggested that a transformation of the data using a log_10_ scale would be appropriate for analysis of significant differences. The log transformation has the added benefit that results can be interpreted as a 10‐fold change. Analysis of variance (ANOVA) was used to examine differences in time and allele and to determine which differences might be significant. These analyses demonstrate higher expression in turning (Breaker +3 d) and ripe (Breaker +7 d) relative to mature green (MG) and breaker (B) fruit (*P* = .004) and higher expression with B alleles (*P* = 7.171 × 10^−7^). The difference between *B* and *b* alleles was maximized at timepoint three and four. At these timepoints, the source of the *B* promoter significantly affected expression (*P* = 1.881 × 10^−6^), with the Jaune Flamme and 97L97 alleles showing higher expression relative to the allele from *S. pennellii* (LA3502) and the allele from OH8245 (Figure 5b). These results were consistent with the carotenoid levels detected in fruit.

## 4 DISCUSSION

Plant germplasm repositories and horticultural seed catalogs contain a wide array of diversity with respect to tomato fruit color. We profiled 29 tomato varieties and determined that of the 20 with orange fruit, nine had elevated β‐carotene levels and 11 varieties contained tetra‐*cis*‐lycopene as the predominate carotenoid. Tetra‐*cis*‐lycopene, a lycopene isomer unique to these tomato fruit, imparts a characteristic tangerine color to tomato (Isaacson, Ronen, Zamir, & Hirschberg, 2002; Zechmeister, 1944). Of the 9 high β‐carotene tomatoes we profiled, three were known to be derived from wide crosses in which the *B* allele imparted orange fruit when introgressed into a red‐fruited genetic background. For two of these varieties, the donor sources were green‐fruited species (*S. chilense* and *S. habrochaites*). The third was derived from the orange‐fruited wild tomato *S. galapagense* native to the Galápagos Islands of Ecuador.

**FIGURE 6:**
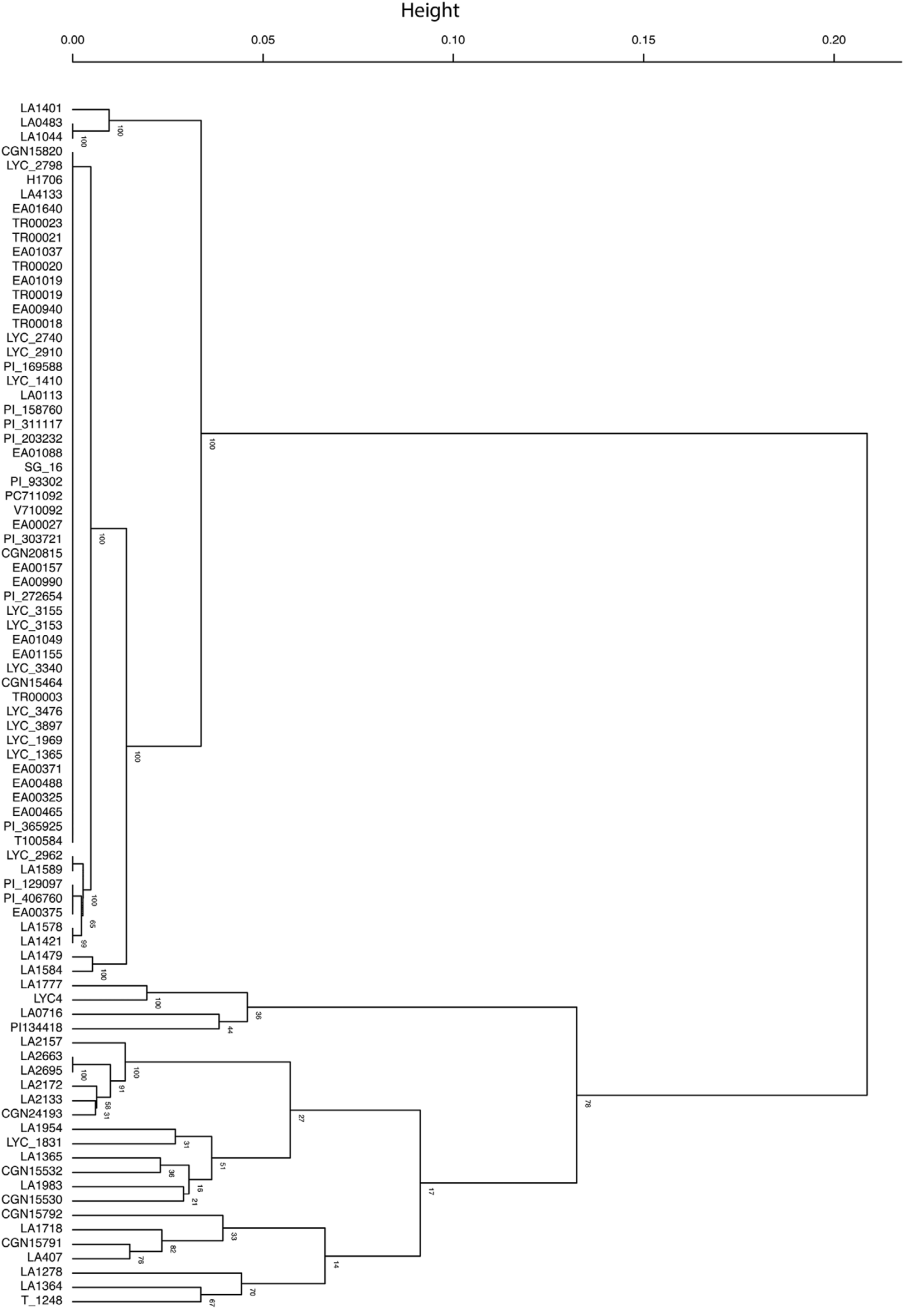
Dendrogram of the *Cyc‐B* promoter region from 84 unique tomato accessions from The 100 Tomato Genome Sequencing Consortium. Nodes represent tomato accessions with overlaid bootstrap values from complete‐linkage hierarchical clustering

Sequence analysis confirmed that the region 5′ to *Cyc‐B* is highly diverse with 30 haplotypes among the 84 unique genomes in The 100 Tomato Genome Sequencing Consortium and 13 identified in the vintage and wild accessions we sequenced (Figures 2 and 6). We observed known polymorphisms in *S. pennellii* and *S. habrochaites* (Dalal et al., 2010; Ronen et al., 2000) and also identified additional variation that was not described previously. Clustering results from this panel of *B* promoter regions suggest that the *B* haplotypes found in vintage cultivars, contemporary breeding lines, and hybrids were originally derived from wild tomato species. Clustering of the Cyc‐*B* promoter sequences implies that the high β‐carotene haplotypes are ancestral to red‐fruited varieties. This origin of haplotypes from wild species is consistent with historical practices of introgression (Lincoln & Porter, 1950; Ronen et al., 2000). Although the origin of some of the sequence variation can be obtained from pedigree records, the source of variation in vintage cultivars is more ambiguous. For example, within the tomato‐seed‐saving community, Jaune Flamme is described as an orange‐fruited heirloom originating from France (Kingsolver et al., 2008). Sequence comparison shows that its promoter is identical to *S. habrochaites* LA0316. This identity suggests that Jaune Flamme derived its *B* allele following introgression from *S. habrochaites* into cultivated germplasm. This introgression may have occurred as early as the 1950s based on work in Indiana using LA0316 (Lincoln & Porter, 1950; Tomes et al., 1954, 1956). For most of the high β‐carotene germplasm we evaluated, the *B* allele appears to have been introgressed from a wild relative, with the possible exception of Western Seed 862978.

The Galápagos Island species, *S. galapagense and S. cheesmaniae*, cluster independently from the red‐fruited accessions. There were five haplotypes, with three identified in multiple accessions. The haplotype found in LA1039 and LA1407 was unique to *S. cheesmaniae* accessions from two islands (Isabela and Fernandina). The haplotype found in LA0438, LA0483, and LA1044 was unique to *S. galapagense* from three islands (Isabela, Fernandina, and Bartolome). The third haplotype was present in accessions of both species that originated from seven islands. The *S. cheesmaniae* accession LA1409 and *S. galapagense* accession LA0526 had unique haplotypes. These results suggest either a single origin of a β‐carotene locus and subsequent divergence on the Galápagos, or separate alleles originating in *S. galapagense* and *S. cheesmaniae* and then subsequent gene exchange between the two species.

Caro Red was the only high β‐carotene cultivar to contain a *B* allele identical to red‐fruited accessions. Previous studies have mentioned the presence of the additional gene *Beta*‐modifier (Mo_B_) that interacts with the *B* allele to influence β‐carotene content (Tomes et al., 1954; Zhang and Stommel, 2000). In the case of Caro Red, this modifier or other unknown genes may be present and cause elevated levels of β‐carotene without a sharp decline in lycopene content.

Association analysis using different statistical models and two populations consistently identified SNPs at −401 and −506 bp as the most significantly associated with high β‐carotene. These two SNPs were also present in *B* loci described in the Ph.D. dissertation of Falcone (2009) and are also found in the *S*. *cheesmaniae* and *S*. *galapagense* accessions included as part of The 100 Tomato Genome Sequencing Consortium (The 100 Tomato Genome Sequencing Consortium et al., 2014) and the Tomato 360 Genome Sequencing Project (Lin et al., 2014), further strengthening the argument that these SNPs represent the most likely candidates for the functional mutations responsible for high β‐carotene. One SNP, located −506 bp from the ATG, is within a putative CAAT box and possibly involved with binding transcription factors (Dalal et al., 2010). Understanding how the two SNPs contribute to phenotypic variation will require further analysis. In addition, more complete analysis will be required to determine whether the multiple other SNPs and indels play a role in modulating the *Beta* phenotype.

The presence of distinct *B* promoter haplotypes not only illustrates the role of wild species in contributing new genetic variation but also suggests the possibility of additional variation within the wild relatives of the cultivated tomato. Breeding efforts involving wild tomato species clearly contributed to sequence diversity 5′ to the *B* coding sequence in cultivated material. Within the subset of *Beta* accessions, we observed a range of β‐carotene levels. It is possible that diverse genetic backgrounds or modifying alleles such as Mo_B_ are responsible for this variation but, also equally likely, that sequence variation directly 5′ to *B* is manifested in the observed phenotypic variation. The mutations identified in this study may be used for marker‐assisted breeding of tomato varieties in order to test this hypothesis. The identification of haplotypes associated with high β‐carotene may provide plant breeders with increased diversity to modulate β‐carotene and carotenoid production through selection. Phenotypic evaluation of our nearly isogenic genetic resources suggests that haplotypes have distinct phenotypes corresponding to different alleles and suggests the possibility of using natural variation to modulate levels of β‐carotene and to gain insight into regulation resulting from sequence diversity.

Theoretical and simulation studies suggest that background genome selection will be effective with few markers and population sizes that are reasonably maintained in a breeding program (Herzog & Frisch, 2011). We adopted this approach in the BC_1_ stage and used single‐marker PCR assays to reduce the number of individuals required for high‐throughput genotyping. The *B* PCR marker that we developed could differentiate the heterozygotes and homozygous alleles of the *B* promoter. The availability of SNP resources (Sim et al., 2012a, 2012b) permitted background genome selection for tomato even though the crosses involved cultivated donor and recurrent parents. Although several thousand SNPs are available throughout the tomato genome, simulations suggest that two to four markers per 100 cM chromosome are sufficient to maintain a high level of background selection efficiency (Hospital, Chevalet, & Mulsant, 1992; Servin & Hospital, 2002; Visscher, Haley, & Thompson, 1996). Increasing marker density beyond this level may not yield significant gains in the ability to recover the recurrent parent genome (Hospital et al., 1992). We used custom sets of 96 and 72 SNP markers to select backcross individuals with the most recurrent parent genetics at the BC_1_ and BC_2_ stages. The use of two donor sources of the *B* allele, M82 and Jaune Flamme, necessitated the average marker number per chromosome for the BC_1_ array to be twice the minimum number recommended in simulation studies because some markers were only polymorphic across a single donor source. In addition, the input requirements for the BeadXpress (Illumina) platform determined that 96 SNPs would need to be used once we determined that 48 SNPs would provide insufficient coverage for the two donor genomes. In the BC_1_ generation, the average recurrent parent contribution for all three populations was ∼75%, matching the expected proportion in a conventional backcrossing scheme. We were able to select individuals containing between 83 and 88% recurrent parent genetics, essentially skipping a generation of backcrossing. These gains were repeated at the BC_2_ stage. After two rounds of foreground and background selection, the final BC_2_ plants were approximately equivalent to BC_4_ individuals, containing between 93 and 98% recurrent parent genetics. We observed expected gains with relatively small population sizes and marker numbers. Targeting specific regions of the genome that were not fixed after initial rounds of background genome selection will allow for reduced marker numbers in later rounds of selection. The alternate approach of saturating these targeted regions with markers, thus increasing the overall marker number, may also allow for efficient recurrent parent genome recovery in later generations. Overall, the results from two rounds of background genome selection confirm that marker‐assisted backcrossing with relatively small marker sets can be used to make generational gains.

There are few empirical examples of background genome selection in tomato. Of these studies, most did not use SNPs and focused on introgressed traits from wide crosses with wild tomato species (Goodstal, Kohler, Randall, Bloom, & Clair, 2005; Lecomte et al., 2004). The present study is one of the first examples demonstrating the practical use of background genome selection in crosses between cultivated germplasm. While the *B* allele was derived from wild species in both Jaune Flamme and the *S. pennellii* introgression lines, the donor genomes were both cultivated. Furthermore, M82, the donor genome in LA3501 and LA3502, is in the same market class as OH8245. Both represent processing tomato germplasm, with OH8245 in the Midwest U.S. clade and M82 in the California clade (Sim et al., 2012b). As SNP resources and genotyping platforms for tomato continue to expand, background genome selection for cultivated crosses will become routine. Even when working with cultivated donor genomes, there are still challenges to introgression assisted through background genome selection. The necrosis observed in the first year field trials in the LA3501 backcross populations may have been due to additional sequence elements associated with LA3501 introgression. A study describing the fine‐mapping of *RXopJ4*, a *S. pennellii* resistance locus for bacterial spot of tomato caused by *Xanthomonas perforans*, observed severe leaf necrosis in populations using LA3501 as a parent, suggesting negative effects from linkage drag (Sharlach et al., 2013).

With respect to the *B* locus, natural variation can be exploited to modulate levels of β‐carotene in ripe tomato fruit. Cultivated varieties with elevated β‐carotene and lycopene content are currently available on the market; however, further modulation of carotenoid content may be required when developing new biofortified foods (Hirschi, 2008; Silletti et al., 2013; Welch, 2002). In addition, plant genetic resources with assorted carotenoid contents can be used to test hypotheses about human adsorption and utilization and metabolism of nutrients in the food matrix (Cooperstone et al., 2015; Kopec et al., 2014).

We introgressed promoter haplotypes for high β‐carotene content into the same cultivated genetic background. Three *B* promoter haplotypes were compared, each with distinct sequence differences from the donor parents. Marker‐assisted backcrossing in two generations led to the selection of plants that shared 93–98% of the recurrent parent and differed in β‐carotene content suggesting the haplotypes represent distinct alleles from *S. galapagense* (*B* ^LA0381^), *S. pennellii* (*B* ^LA0716^), and *S. habrochaites* (*B* ^JF^). Field trials containing progeny from these selections showed that modulation of β‐carotene content could be achieved through allele choice. The state of the *B* allele (*bb, Bb, BB*) accounted for the most variation in β‐carotene content, because plants with the wild‐type allele (*bb*) do not exhibit increased cyclization of lycopene by lycopene‐β‐cyclase (Ronen et al., 2000). Results from replicated field trials support the classical definition of *B* as semidominant. Levels of β‐carotene in heterozygotes were nearly as high as those found in homozygous *BB* plants. Interestingly, BC_1_S_1_ family within population explained ∼15% of the total variation in β‐carotene content. However, following selection and crossing, this variation was reduced to 0.65% in the 2‐yr dataset. Backcrossing the BC_1_ selections to form the BC_2_ populations may have removed other influential loci outside of the *B* locus. The donor source of the *B* allele was more important than family within a specific source, demonstrating that the three sources (*B* ^LA0716^, *B* ^LA0381^, and *B* ^JF^) had the effect of modifying β‐carotene levels. This result suggests that diversifying donor sources of the *B* allele can also diversify the concentration of β‐carotene.

Fine tuning of carotenoid content in tomato is possible using natural allelic variation. The findings of the present study have implications for the development of functional foods with various carotenoid profiles. This study also provides a model for rapidly introgressing natural variation into an elite background and assessing the effects of that variation. As plant breeders continue to fix beneficial alleles for traits in crops, new allelic variation will be required in order to provide further enhancements for important traits.

## AUTHOR CONTRIBUTIONS

Jessica L. Cooperstone, Formal analysis, Investigation, Editing. Elisabet Gas‐Pascual: Formal analysis; Investigation; Methodology; Writing‐review & editing. Gabriel Abud: Data curation; Formal analysis; Software. Steven J. Schwartz, Investigation. Caleb Orchard and David M. Francis, Methodology, Formal analysis, Investigation, Writing‐Review & editing.

## CONFLICT OF INTEREST STATEMENT

The authors declare that they have no competing interests.

## Data Availability

Sequences generated for these studies have been deposited into the National Center for Biotechnology Information (NCBI) and assigned GenBank Submission Numbers KP233148‐ KP233173.
